# Hepatosplenic Bartonellosis in an Immunocompetent Teenager: An Atypical Presentation of Cat-Scratch Disease

**DOI:** 10.7759/cureus.13219

**Published:** 2021-02-08

**Authors:** Rohit Sharma, Abdullah Mohammad Arshad, Sundus Sardar, Abdulaziz Zafar

**Affiliations:** 1 Internal Medicine, Hamad Medical Corporation, Doha, QAT

**Keywords:** hepatosplenic bartonellosis, visceral bartonellosis, cat scratch disease, fever of unknown origin

## Abstract

Infection with* Bartonella henselae*,a gram-negative coccobacillus, most frequently presents as cat-scratch disease (CSD) and often accompanies a recent history of cat bite or scratch. As compared to adults, teenagers and children or immunocompromised patients are predominantly affected by CSD. In immunocompetent individuals, CSD is typically a self-limiting clinical syndrome with complete resolution of febrile illness in two to four weeks with or without antimicrobial therapy. While most cases present with fever of unknown origin (FUO), previous reports have also documented atypical clinical presentation or systemic symptoms in few cases, including reports of hepatosplenic involvement. We present a case of visceral bartonellosis in an immunocompetent 15-year-old female, who presented with a six-week history of fever and abdominal pain with hepatosplenomegaly. She recovered completely after prolonged antibiotic treatment for six weeks with doxycycline and amikacin. We emphasize that in the workup of FUO, it may be pertinent to include bartonellosis as a differential especially in cases exhibiting hepatosplenomegaly on examination along with hepatosplenic lesions on imaging.

## Introduction

Cat-scratch disease (CSD) caused by *Bartonella henselae*,* *is an infectious disease transmitted by exposure to cats, especially those infested with fleas. The disease is limited to regional lymphadenopathy but dissemination to liver, spleen, eye, bone, and central nervous system may occur. Localized disease is typically self-limiting while the disseminated disease may lead to life-threatening complications. Probable diagnoses can be made by a reported exposure to a cat, and clinical findings which can be further confirmed by serological tests.

Physicians routinely encounter fever of unknown origin (FUO). Keeping in mind, a wide differential and thorough workup are essential to come up with a definitive diagnosis and treatment. We report a case of a 15-year-old female, who presented with fever and abdominal pain of more than six-week duration, who was diagnosed with visceral bartonellosis (CSD). Imaging investigations revealed hepatosplenomegaly with multiple hepatic and splenic lesions. The patient reported recent exposure to an ill cat and *B. henselae* was suspected based on presentation and epidemiological link. She was treated with prolonged therapy of amikacin and doxycycline, which resulted in clinical as well as radiological improvement. Hepatosplenic complications of CSD are rare in immunocompetent individuals [1]. Only a few cases reported so far have presented with fever and abdominal pain [1].

## Case presentation

Clinical findings

We present the case of a 15-year-old female, with a past medical history of asthma, who presented with a six-week history of high-grade fever and left flank pain. She reported being febrile mostly every night, with temperature readings reaching up to 40ºC as recorded at home. Her fever responded to antipyretics and was associated with malaise, fatigue, and generalized body ache. She denied any history of chills, sweating, or significant weight loss. She was noted to have dull, intermittent, left-sided non-radiating flank pain of moderate intensity with no specific aggravating or relieving factors and not associated with vomiting or any changes in bowel habits. She had recently returned from Jordan and also complained of dry cough for one month. She denied lymphadenopathy, history of sick contact, rashes, shortness of breath, joint pain, or swelling. Family history was significant for pancreatic malignancy in the father but not for any rheumatological disorder. On detailed history taking, the patient mentioned exposure to a cat one week prior to the onset of symptoms. The cat was owned by a friend and subsequently became ill to a point that the animal was treated by a veterinary doctor. The outcomes of the cat are unknown. The initial assessment revealed a temperature of 38.7ºC, pulse of 124 beats per minute, blood pressure of 119/73 mmHg, respiratory rate of 18/min, and oxygen saturation of 99% on room air. Physical examination was significant for hepatomegaly with liver edge palpable 2 cm below the coastal margin and no lymphadenopathy. The rest of the examination and review of systems were all unremarkable.

Diagnostic assessment

Initial laboratory investigations revealed microcytic anemia, high erythrocyte sedimentation rate (56 mm/hr), high C-reactive protein (CRP) and normal procalcitonin, mild hypercomplementemia (C3 1.96 g/L and C4 0.44 g/L), and negative antinuclear antibodies (ANA), and anti-neutrophil cytoplasmic antibodies (ANCA) (Table 1). 

**Table 1:** Laboratory investigations WBC, white blood cell; MCV, mean corpuscular volume; ESR, erythrocyte sedimentation rate; CRP; C-reactive protein; ANA, anti-nuclear antibodies; ANCA, anti-neutrophil cytoplasmic antibodies; CCP, cyclic citrullinated peptide; DsDNA, double-stranded DNA; IgG, immunoglobulin G; IgM, immunoglobulin M; EBV, Epstein-Barr virus; Ag/Ab, antigen/antibodies

Laboratory investigations	Value	Lab reference
WBC	4.8 x 10^3^/uL	4.0-10 x 10^3^/uL
Hemoglobin	9.5 gm/dL	12-15 gm/dL
Platelets	424 x 10^3^/uL	150-400 x 10^3^/uL
MCV	75.1 fL	83.0-101.0 fL
ESR	56 mm/hr (high)	2-37 mm/hr
Haptoglobin	411 mg/dL	30-200 mg/dL
CRP	86.5 mg/L	0-5 mg/L
Procalcitonin	0.07 ng/mL	<0.5 ng/mL represents a low risk of sever sepsis and/or septic shock; >2.0 ng/mL represents a high risk of sever sepsis and/or septic shock
Rapid malaria test	Negative	
ANA	Negative	
ANCA	Negative	
Anti-CCP Ab	Negative	
Anti-DsDNA Ab	Negative	
C3	1.96 gm/L	0.62-1.2 gm/L
C4	0.44 gm/L	0.15-0.41 gm/L
Quantiferon TB	Negative	
Brucella IgG and IgM	Negative	
Leishmania Ab	Negative	
Cytomegalovirus IgG	Reactive	
Cytomegalovirus IgM	Non-reactive	
Toxoplasma Ab IgG	Reactive	
Toxoplasma Ab IgM	Non-reactive	
EBV capsid antigen IgG and IgM	Negative	
Hepatitis B surface antigen	Negative	
Hepatitis C antibody	Non-reactive	
HIV Ag/Ab combo	Non-reactive	
*Bartonella henselae* IgG	<1:128 titer	
*Bartonella henselae* IgM	<1:20 titer	
*Bartonella quintana* IgG	<1:128 titer	
*Bartonella quintana* IgM	<1:20 titer	

Further investigations for tuberculosis including acid-fast bacilli smear and polymerase chain reaction (PCR) were negative from sputum and bone marrow. Culture from blood, sputum, bone marrow, and urine yielded no growth. A total of seven sets of blood culture (aerobic and anaerobic) were negative starting from the day of admission through day 5. Serum immunoglobulin G (IgG), IgM, and IgA values were within normal limits. Flow cytometry and bone marrow biopsy were negative for hematological malignancy. Kidney function, liver functions, electrolytes, lipid profile, and HbA1c were normal.

Radiology

**Figure 1:**
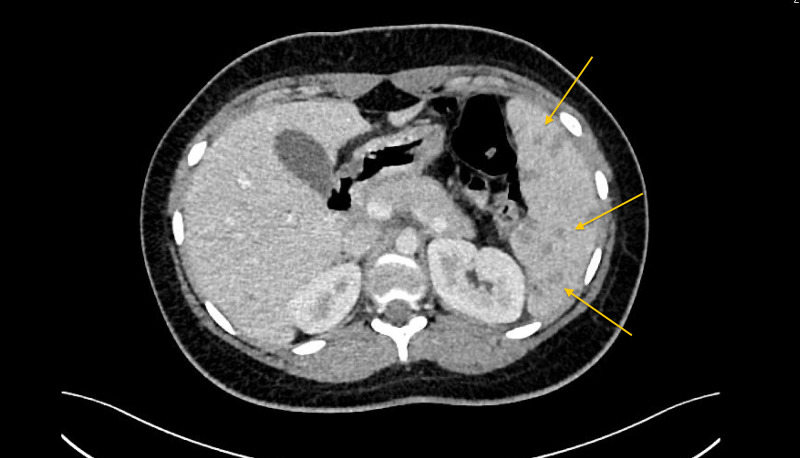
CT scan of the abdomen showing multiple hypodense lesions in the spleen (marked by yellow arrows)

**Figure 2:**
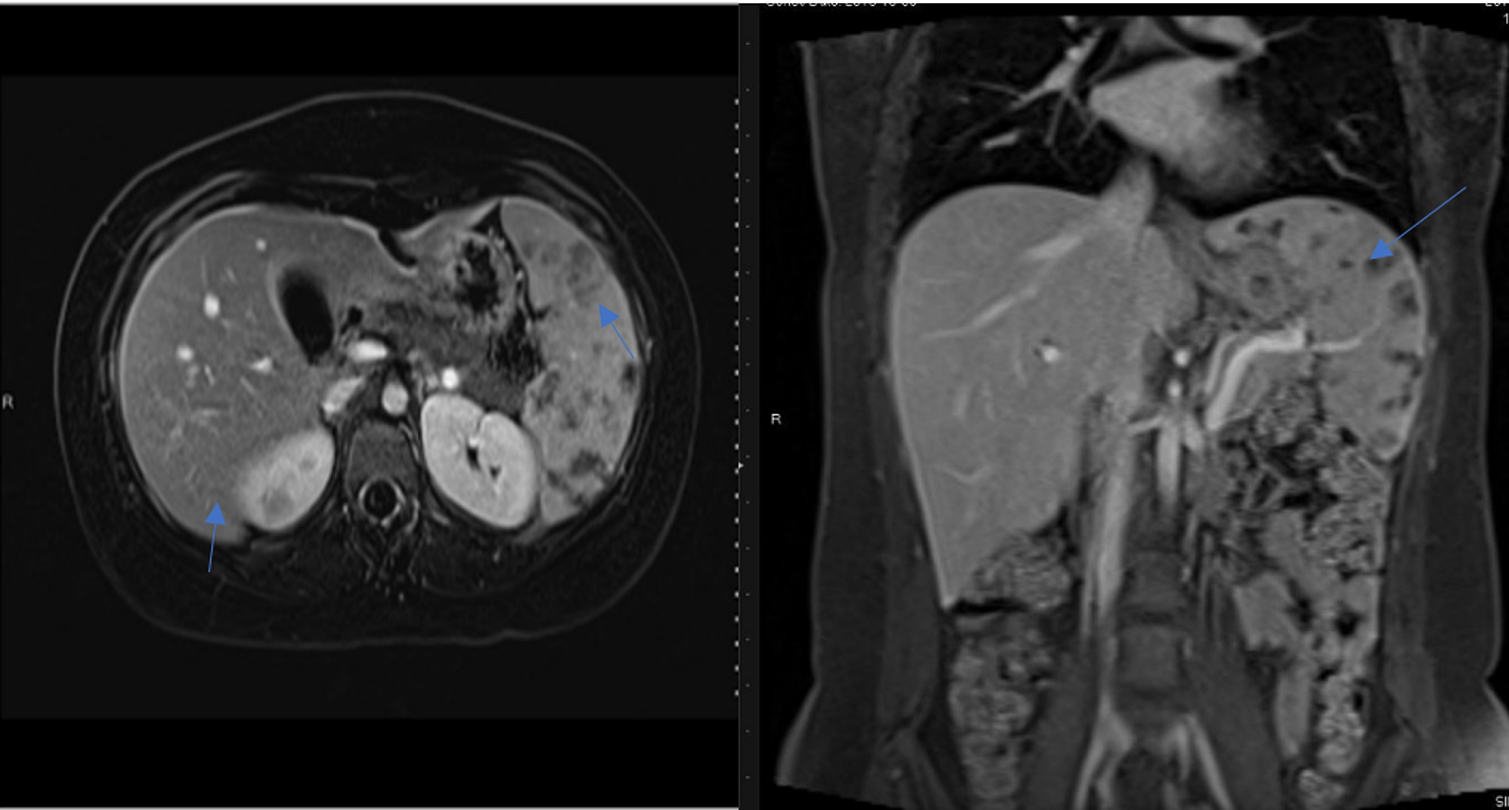
MRI of the abdomen - T1 weighted post-contrast transverse view (left image) and coronal view (right image) showing multiple hypodense lesions in the liver and spleen (marked by blue arrows)

**Figure 3:**
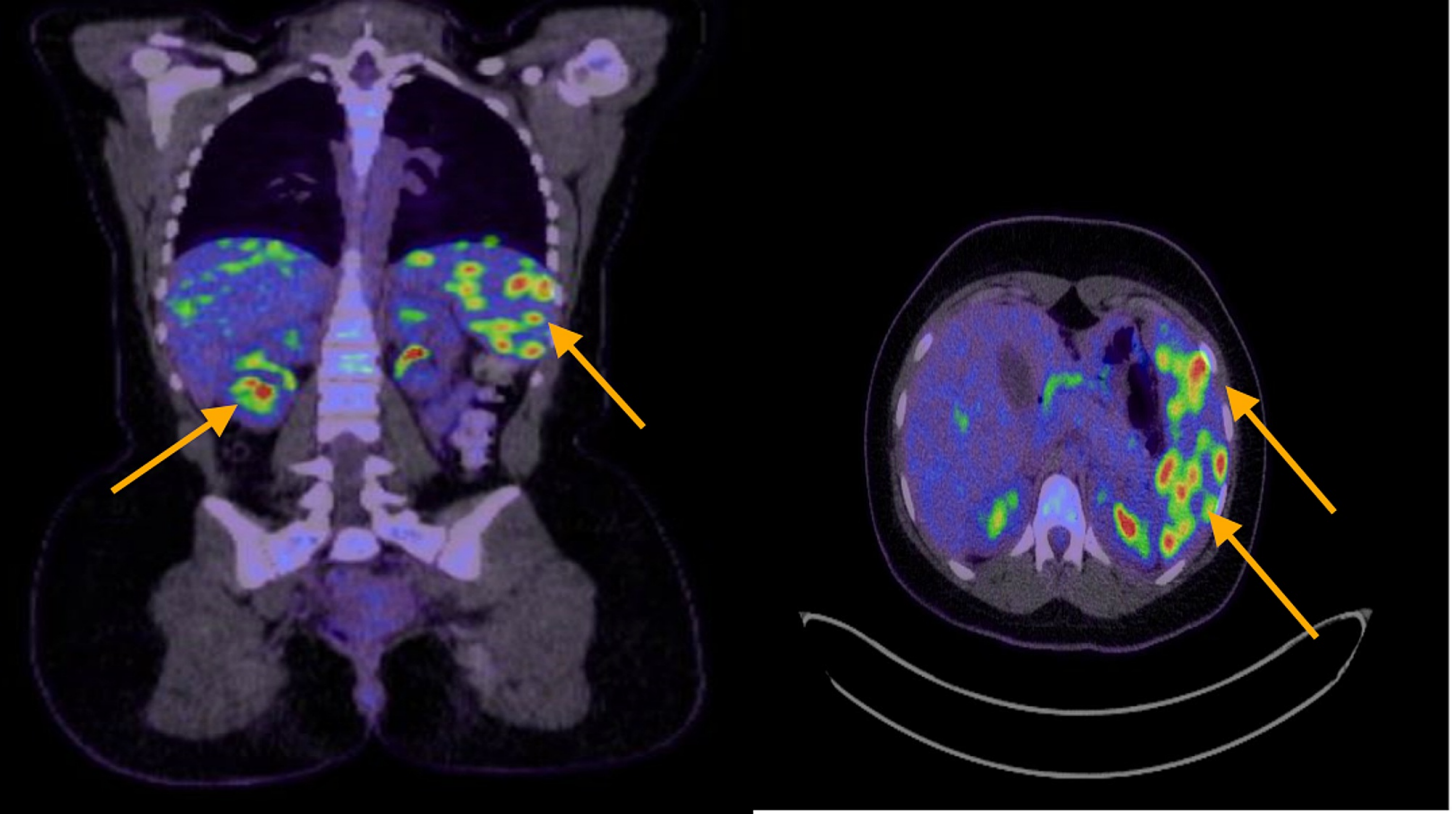
PET FDG scan showing increased multiple focal uptakes in the liver and spleen (marked by yellow arrows) PET, positron emission tomography; FDG, fluorodeoxyglucose

The chest x-ray was unremarkable. Ultrasound of the abdomen confirmed mild hepatomegaly and splenomegaly with multiple ill-defined hypoechoic lesions in the spleen. Computed tomography (CT) of the abdomen revealed hepatomegaly measuring 19.5 cm and splenomegaly measuring 10 x 13.5 cm with multiple variable-size hypodense lesions with no wall enhancement (Figure 1). Results from magnetic resonance imaging (MRI) of the abdomen showed hepatosplenomegaly, multiple splenic and small ill-defined hepatic lesions, and a right renal lesion (Figure 2). Whole-body positron emission tomography (PET) revealed intense hypermetabolism in multiple splenic and liver lesions (Figure 3). A transesophageal echocardiogram was negative for any masses or vegetation. Biopsy of the identifed lesions was not obtained.

Therapeutic intervention

The patient was initially started on ceftriaxone, vancomycin, and meropenem upon admission. Upon presumptive diagnosis of bartonellosis, 10 days after admission, the antimicrobial coverage was changed to amikacin for a duration of two weeks and doxycycline for six weeks.

Follow-up and outcome

**Figure 4:**
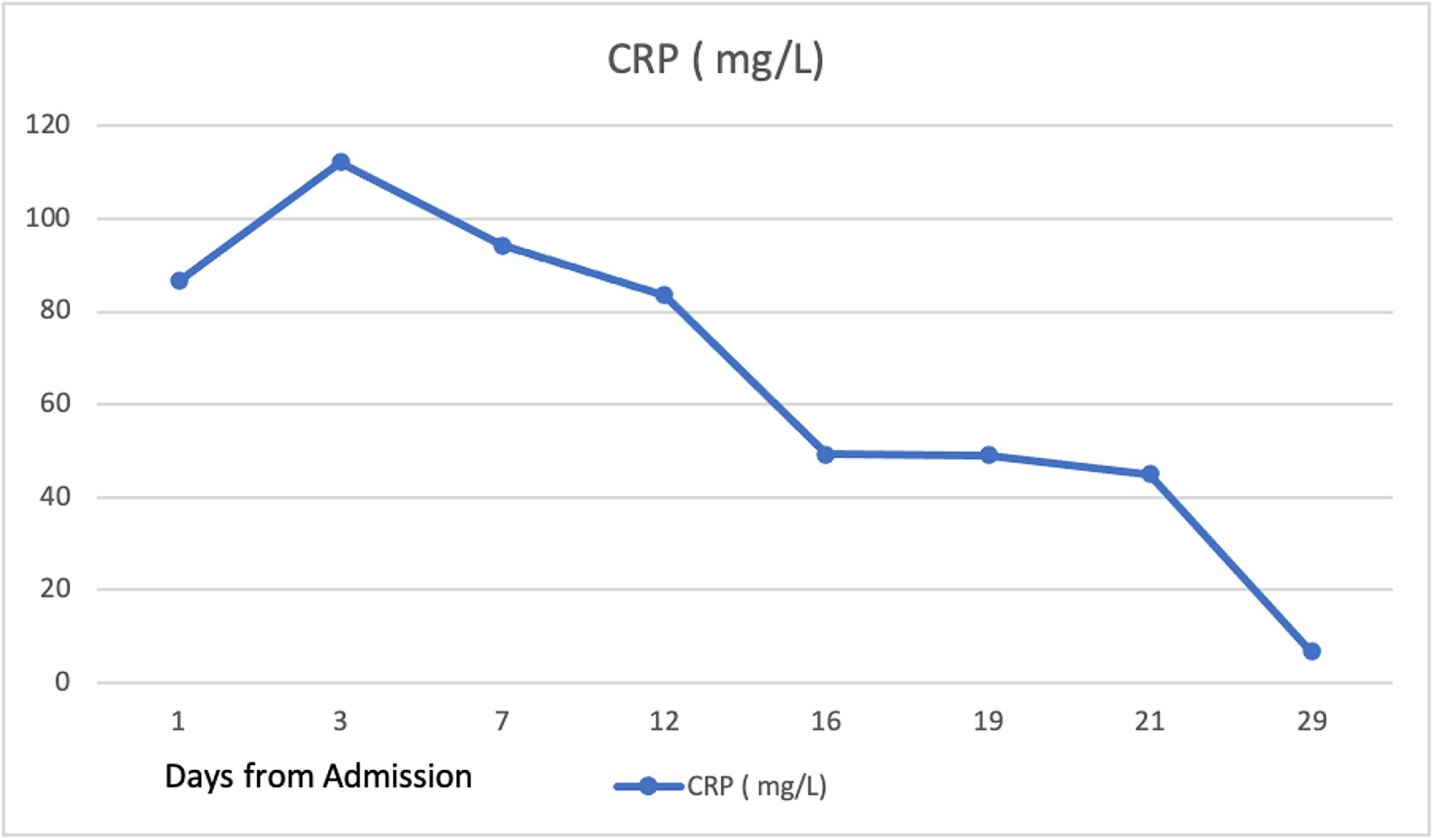
Trend of CRP with treatment against visceral bartonellosis CRP, C-reactive protein

After initiation of amikacin and doxycycline therapy directed against visceral bartonellosis, the patient showed significant clinical improvement in terms of fever and abdominal pain. CRP values continued to improve with treatment (Figure 4). A follow-up MRI of the abdomen two weeks after starting treatment demonstrated hepatosplenomegaly with multiple splenic lesions, which were regressing in size. The previously noted small ill-defined hepatic lesions and the right renal lesion had resolved completely. Findings were impressive for the regression of the disease, thereby supporting our diagnosis of visceral bartonellosis.

## Discussion

CSD is an infectious disease mostly caused by *B. henselae*, a facultative intracellular Gram-negative coccobacillus, with a worldwide distribution [2]. Very rarely CSD may be caused by *Afipia felis*, *Bartonella clarridgeiae*, or unidentified fastidious organisms [3,4]. Pathogenesis of the disease remains unclear and manifestations are either local (cutaneous, regional lymphadenopathy) or systemic (ocular, visceral, neural, or musculoskeletal involvement) [5]. Cats are the primary and natural reservoir, often reported to have persistent intraerythrocytic bacteremia sometimes lasting for more than a year [6]. It is typically caused by a scratch or bites from a cat or exposure to cat fleas, usually leading to a localized cutaneous lesion or self-limiting regional lymphadenopathy [5]. Bartonella causes endothelial cell disruption, activation of a proinflammatory cascade leading to acute inflammation, subsequently followed by a rise in inflammatory markers [7]. Although transmission primarily occurs through the infected cat, however, in some cases, fleas or possibly dogs infested with fleas may also be implicated [8].

Visceral organ involvement is rare in CSD. Most of the data available are from the pediatric age group. Hepatosplenic bartonellosis, as in our case, is a rare entity in immunocompetent individuals. As far as adults are concerned, only a few cases of CSD with hepatosplenic involvement in immunocompetent individuals have been reported [1]. When relevant, CSD must be considered in the differential diagnosis of FUO. Probable diagnosis can be made by history and presentation of typical features. Serological testing such as enzyme immunoassay or indirect fluorescence assay is useful in confirming the diagnosis [9,10]. A negative serological test cannot rule out CSD in highly suspected cases. The recommendation is to do a paired serological test, which was unfortunately not done in our case due to the lack of availability. Lymph node or tissue biopsy may be performed in certain circumstances to confirm the diagnosis or when alternative diagnoses such as tuberculosis or lymphoma are suspected. A biopsy may reveal necrotizing granulomatous inflammation, and Warthin-Starry stain may demonstrate pleomorphic *B. henselae* bacilli [5]. Imaging techniques are needed to diagnose visceral involvement in CSD. Ultrasound, CT, and MRI may be useful for the assessment of hepatic, splenic, or renal involvement. In our case, we performed an ultrasound, CT, MRI as well as a PET scan to assess the degree of visceral involvement. Imaging may also be helpful to follow up and evaluate the clinical response to treatment. We appreciated a significant radiological resolution of intra-abdominal lesions in our case. Treatment of hepatosplenic CSD and FUO is with azithromycin plus rifampin; alternatively, rifampin and gentamycin can be used [11-13]. High-dose azithromycin (500 mg PO/IV for five days) alone could be used for immunocompetent adults [1]. Our case was treated with two weeks of amikacin and six weeks of doxycycline to potentially cover *Chlamydia psittaci* or other atypical infections although visceral bartonellosis was most likely the cause based on presentation, epidemiological link, and response to treatment.

Currently, no specific markers or titers are available for monitoring patient’s responses to treatment. We monitored the response to treatment with CRP and follow-up abdominal imagining. Although further imaging may not be required, follow-up imaging at six months after the completion of treatment may be warranted to document regression or resolution of lesions.

## Conclusions

CSD caused by *Bartonella henselae* is an important differential while evaluating for fever of unknown origin. Hepatosplenic complications may occur, although rare in immunocompetent individuals. Advanced imaging, and Bartonella serology coupled with detailed history maybe useful in diagnosis of such cases.
